# Effect of probiotics on glycemic control and lipid profiles in patients with type 2 diabetes mellitus: a randomized, double blind, controlled trial

**DOI:** 10.3389/fendo.2024.1440286

**Published:** 2024-09-16

**Authors:** Xuchao Peng, Hong Xian, Ning Ge, Lisha Hou, Tianjiao Tang, Dongmei Xie, Langli Gao, Jirong Yue

**Affiliations:** Department of Geriatrics and National Clinical Research Center for Geriatrics, West China Hospital of Sichuan University, Chengdu, China

**Keywords:** probiotics, lipid profile, type 2 diabetes mellitus, randomized controlled trial, glycemic

## Abstract

**Introduction:**

This double-blind, placebo-controlled, randomized (1:1) clinical trial was conducted at the West China Hospital, Sichuan University, from March to September 2017.

**Methods:**

Eligible participants included adults aged 18 years and older, living in the community, diagnosed with type 2 Diabetes Mellitus according to ADA guidelines, capable of self-managing their diabetes, and able to visit the study site for follow-up. The intervention group received 25 ml of a probiotic beverage containing with over 10^8 CFU/mL of Lactobacillus, administered four times daily. An equal volume of inactivated Lactobacillus was administered to the control group and the control group was administered the same volume of inactivated Lactobacillus. This study aimed to evaluate the effectiveness of probiotics on glycemic control and other diabetes-related outcomes in patients with type 2 diabetes patients. The primary outcomes were changes in HbA1c and FBG levels post-intervention. Investigators, participants, and study site personnel were blinded to the treatment allocation until the conclusion of the study. This double-blind, randomized, placebo-controlled clinical trial was registered in the Chinese Clinical Trial Registry (ChiCTR-POR-17010850).

**Results:**

Of the 490 participants screened, 213 were randomized to either the probiotics group (n = 103) or the placebo group (n = 110). After 16 weeks of follow-up, the probiotic group showed reductions in HbA1c [-0.44 (-0.66 to -0.22)] and FBG [-0.97 (-1.49 to 0.46)] post-intervention, similar to the placebo group with reductions in HbA1c [-0.33 (-0.52 to -0.15)] and FBG [-0.90 (-1.32 to -0.47)], but these changes were not statistically significant in PP and ITT analyses (P>0.05). Adverse events were similarly distributed among groups, indicating comparable safety profiles.

**Discussion:**

Overall, 16-week probiotic supplementation showed no beneficial effects on glycemic control, lipid profiles, or weight.

**Clinical Trial Registration:**

https://www.chictr.org.cn/showproj.html?proj=18421, identifier ChiCTR-POR-17010850.

## Introduction

Type 2 diabetes mellitus (T2DM) is a chronic metabolic disorder characterized by elevated blood glucose levels owing to inadequate insulin production and insulin resistance. The International Diabetes Federation (IDF) has projected that the prevalence of T2DM will increase to 629 million by 2045 (1). This condition is associated with numerous complications, such as nephropathy, retinopathy, atherosclerosis, and coronary heart disease, significantly contributing to the global burden of mortality and morbidity (2).

The human gut microbiota, comprising trillions of microorganisms across at least thousand different species, including *Bifidobacteria*, *Lactobacillus*, and *Saccharomyces* (3), plays a pivotal role in food digestion and absorption, immune regulation, pathogen resistance, and synthesis of beneficial compounds (4, 5). Alterations in the composition and functionality of the gut microbiota can affect energy extraction, lipid synthesis, and energy metabolism shifts, potentially leading to metabolic syndromes (6). Moreover, accumulating evidence underscores the critical role of the gut microbiota in the pathogenesis of T2DM (7–10). Probiotics can be administered orally, transiently, or permanently to integrate and interact with intestinal microbiota, thereby influencing host health (11). Recent studies have highlighted the antidiabetic potential of probiotic supplementation, potentially enhancing glucose regulation, lipid metabolism, antioxidant capacity, and modulation of gut flora and SCFA profiles (12, 13). Several studies have demonstrated the potential of probiotics to improve glycemic control in animal models and human trials. In mice fed a high-fat diet, *Lactobacillus plantarum DSM 15313* supplementation lowered fasting plasma glucose levels and reduced insulin release during the glucose tolerance tests (14). Similarly, a mixture of *Lactobacillus rhamnosus* and *Lactobacillus helveticus* decreased blood glucose levels and improved glucose tolerance in a mouse model of metabolic syndrome (15). *Lactobacillus casei* significantly reduced blood glucose levels in diabetic mice without affecting control mice (16). Probiotics have shown promising effects on the glycemic control and lipid profiles of patients with type 2 diabetes. Multiple studies have reported significant reductions in HbA1c and fasting insulin levels following probiotic supplementation (17, 18). Fasting plasma glucose levels were also found to decrease significantly in some trials (18, 19). Regarding lipid profiles, probiotics have demonstrated potential in lowering total cholesterol, triglycerides, and LDL-cholesterol, while increasing HDL-cholesterol (19). Additionally, probiotic supplementation has been associated with reductions in both systolic and diastolic blood pressure (19). Some studies also observed trends towards decreased inflammation markers and oxidative stress, although these findings were not always statistically significant (20). The existing research is primarily limited by small cohort sizes and brief intervention periods; however, these results are encouraging. Researchers have emphasized the need for larger, well-designed, randomized controlled trials to confirm the efficacy of probiotics in managing diabetes and its associated complications (17, 18). In addition, inactivated *Bifidobacterium longum* significantly decreased demonstrated significant effects in decreasing body weight gain, adipose tissue mass, and blood glucose levels in obese diabetic mice (21). Investigating the comparative effects of inactivated and active probiotics on metabolic functions in patients with diabetes may offer novel insights for medical interventions aimed at controlling blood glucose and lipid levels in this population.

Considering these factors, we designed a randomized controlled trial using inactivated probiotics as controls. This trial evaluated the efficacy of fermented drinks containing *Lactobacillus* in improving glycemic control and other diabetes-related outcomes in T2DM patients. This study aimed to investigate the potential therapeutic benefits of probiotics for managing type 2 Diabetes Mellitus (T2DM). We defined our primary outcome as changes in HbA1c and fasting blood glucose (FBG) levels. The secondary outcomes included changes in insulin resistance markers, lipid profiles, and body weight. Safety outcomes were also assessed.

## Subjects and methods

### Study design

We conducted a double-blind, randomized, placebo-controlled clinical trial at West China Hospital, Sichuan University, from March to September 2017 (Chinese Clinical Trial Registry identifier ChiCTR-POR-17010850). The study was approved by the Institutional Review Boards of West China Hospital, Sichuan University, and adhered to the Declaration of Helsinki, Council for International Organizations of Medical Sciences International Ethical Guidelines, and Good Clinical Practice Guidelines. Informed consent was obtained from all participants or their legal representatives before the trial commencement.

### Participants

Eligible participants were community-dwelling adults aged 18 years or older, diagnosed with type 2 Diabetes Mellitus according to the ADA guidelines, capable of self-managing their diabetes care, and able to access the study site for necessary follow-up. The exclusion criteria were as follows: a) a terminal condition with a life expectancy of less than 6 months (e.g., metastatic cancer, pancreatic cancer, or receiving end-of-life care); b) severe renal dysfunction (glomerular filtration rate (GFR) of less than 30 milliliters per minute per 1.73 square meters) or liver dysfunction (significantly elevated serum bilirubin levels (more than twice the upper limit of normal) and markedly prolonged prothrombin time); c) a documented history of alcohol abuse or drug addiction; d) psychiatric disorders preventing completion of geriatric assessments, and pregnant or breastfeeding women;​​ and e) use of any other kind of probiotics or antibiotics in the previous 3-6 months. The use of antibiotics not only interferes with the action of probiotics but also makes blood glucose control more difficult in diabetic patients (22, 23).

### Randomization and blinding

Participants were randomly assigned (1:1 ratio) to either the intervention or the control group using a computer-generated scheme. The intervention group was administered 25 ml of a probiotic beverage, containing over 10^8 CFU/mL of Lactobacillus, four times daily (probiotic group). The control group was administered an equal volume of inactivated Lactobacillus (placebo group). Both probiotic and placebo products were identical in appearance, taste, and smell to ensure blinding of participants and study staff, distinguished only by coded labels (“A” or “B”). All participants were requested to maintain their usual diet, physical activity, and glucose-lowering therapy throughout the study period. The investigators, participants, and study site personnel were blinded to treatment allocation until the end of the study.

### Intervention

The study included a 2-week screening period and 16-week treatment period. During treatment, patients either received 25 ml of probiotic beverage containing more than 10^^8^ CFU/mL of Lactobacillus four times daily (probiotic group) or an equivalent volume of inactivated Lactobacillus (placebo group). Measurements were taken at baseline and at week 16, with an interim analysis at week 8​​.

### Assessments

Following baseline inclusion, all patients underwent evaluations that included collection of sociodemographic data, diabetes history, and diabetes medication history, such as age, sex, and body weight. Binary variables were used to record whether participants were taking metformin, sulfonylureas, gliclazide, or other medications. Body weight was accurately measured to the nearest 0.1 kg using a calibrated digital scale. Blood pressure readings were obtained using an automated upper arm blood pressure monitor.

During the clinic visits (at baseline, week 8, and week 16), fasting blood samples were collected for comprehensive biochemical analyses. Baseline screening and evaluation processes are summarized in sTable1. These included measurements of glycosylated hemoglobin (HbA1c) by TOSOH HLC-723(use of a negatively charged column and positively charged buffers that compete with the different glycosylated hemoglobin to bind to the column), fasting blood glucose (FBG), serum insulin, serum C-peptide-peptide, and a full lipid profile encompassing low-density lipoprotein (LDL), high-density lipoprotein (HDL), very-low-density lipoprotein (VLDL), total cholesterol (TC), and triglycerides (TG), measured using an Olympus AU400 analyzer (Japan)(Spectrophotometry). Insulin resistance was quantitatively assessed using the Homeostasis Model Assessment of Insulin Resistance (HOMA-IR), calculated from fasting glucose and C-peptide-peptide levels using the HOMA calculator version 2.2.3 from the University of Oxford, UK (24). All blood tests were done at West China Hospital of Sichuan University

Intervention compliance was assessed primarily through two methods: (1) Weekly telephone follow-ups: During these calls, we directly asked participants about their adherence to the intervention protocol. (2) Self-reported adherence: Participants were asked to keep a diary of their medication intake, which was reviewed during follow-ups. These methods allowed us to regularly monitor and assess participant compliance throughout the study.

Adverse events were systematically recorded at each follow-up visit. Commonly anticipated adverse events include gastrointestinal disturbances such as diarrhea, dyspepsia, nausea, vomiting, abdominal cramping, and distension. Participants were instructed to discontinue the supplement intake if they encountered any serious adverse events attributable to the intervention.

### Outcomes

The primary outcome of the study was the change in HbA1c and FBG levels from baseline to week 16. Secondary outcomes included changes in HOMA-IR, serum insulin, C-peptide-peptide levels, lipid profiles (LDL, HDL, VLDL, TC, and TG), and body weight from baseline to week 16. Safety outcomes included adverse events, alterations in laboratory parameters, and changes in vital signs and signs.

### Sample size

The required sample size was deduced from prior meta-analyses examining the impact of probiotics on glycemic control in T2DM, which reported a mean HbA1c change of 0.366 (25). Assuming a standard deviation of 0.7, a Type I error rate of 5% (α=0.05), and a Type II error rate of 20% (β=0.2, power=80%), the estimated sample size was 140 participants (70 per group). The final sample size was adjusted to 168 participants (84 per group), to accommodate a projected dropout rate of 20%.

### Statistical analysis

Data were analyzed using SPSS Statistics version 26 (IBM, NY, USA). The Kolmogorov-Smirnov test confirmed the normal distribution of the dataset. Continuous variables are presented as means (standard deviations) for normally distributed data or medians (interquartile ranges) for skewed data. Categorical variables are described as frequencies (percentages). For variables that met the normality assumption, we used parametric tests such as t-tests or ANOVA for comparisons. For variables that did not meet the normality assumption, we employed non-parametric alternatives such as Mann-Whitney U test or Kruskal-Wallis test, and the Pearson Chi-square test for categorical variables. Statistical significance was set at a p-value less than 0.05, which was considered statistically significant.

Differences in outcomes from baseline to week 16 were analyzed using Intention-to-treat (ITT) approaches. ITT analysis included all participants who received at least one dose of the study medication, with missing values imputed via multiple imputations using predictive mean matching. To account for missing values and correlations among repeated measurements, we used weighted generalized estimating equations (WGEE) (Stata 17.0) to compare metabolic parameters (HbA_1c_, FBG, fasting C-peptide-peptide, HOMA-IR, TG, TC, LDL, HDL, VLDL, and weight) among the groups. Assessments conducted at baseline and weeks 8 and 16 were included as outcomes in the WGEE analysis. The covariates were age, sex, use of specific medications (insulin, metformin, sulfonylurea/glinide, α-glycosidase preparations), smoking, and drinking. The group-by-time interaction, which indicates the difference in a given outcome between interventions over time, was considered the primary measure of the intervention effect. This measure allowed us to assess how the effectiveness of the interventions varied across different time points, providing a comprehensive understanding of the changes induced by each intervention over the study period.

## Results

**Figure 1:**
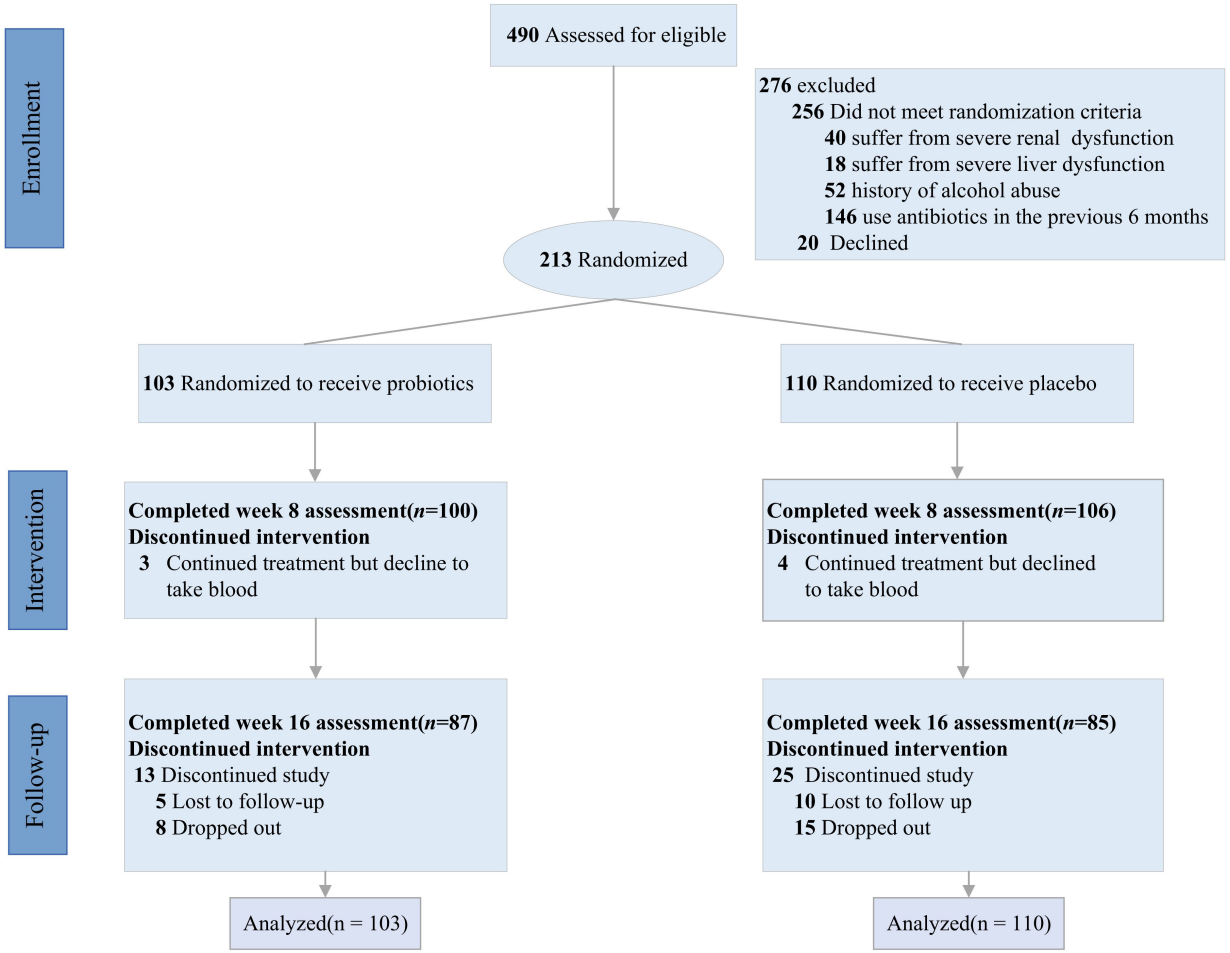
CONSORT flow study diagram.

Out of 490 participants screened for eligibility after providing informed consent, 213 individuals were randomized to either the probiotics group (n = 103) or the placebo group (n = 110). The exclusion of 256 participants was due to not meeting the eligibility criteria: 40 suffered from renal dysfunction, 18 suffered from severe liver dysfunction, 52 had a history of alcohol abuse, 146 had used antibiotics in the previous 6 months, and 20 older declined. Among the 213 participants, 206(100 in the probiotic group and 106 in the placebo group) completed the assessment at week 8, and 172(87 in the probiotic group and 85 in the placebo group) completed the assessment at week 16. Measured as class attendance, were similar (84.5% and 77.3%, respectively) detail in **Figure 1**.

### Baseline characteristics of participants

At baseline, the average age of the participants was 62.08 (SD 10.08) years, with a predominance of male participants (121; 59.6%). The mean weight was 66.4 (SD 11.6) kg. Regarding diabetes management, 65 (31%) participants used insulin, 122 (57%) used metformin, and 68 (32%) used sulfonylurea/glinides. Median baseline HbA1c was 7.20% (IQR 6.54-8.41), and FBG was 8.46 mmol/L (IQR 7.11-10.26). Statistical analysis confirmed that the baseline characteristics were balanced across both groups, with the exception of a higher proportion of metformin users in the placebo group (**Table 1**).

**Table 1:** Baseline characteristics of randomized participants in each group.

Baseline parameter	Probiotics group (n=103)	Placebo group (n=110)	*P* value
Age, mean (SD), y	62.14 (10.25)	61.98 (10.06)	0.91^a^
Male gender, No. (%)	62 (60.2)	65 (59.1)	0.87^c^
Weight, mean (IQR), kg	65.0 (59.0-75.0)	65.0 (59.0-73.0)	0.93^b^
Smoking, No (%)	37 (36.0)	42 (38.2)	0.73
Drinking, No (%)	32 (31.1)	36 (32.7)	0.80
Background diabetes therapy, No. (%)			
Insulin	31 (30.1)	34 (30.9)	0.90^c^
Metformin	50 (48.5)	72 (65.5)	0.01^c^
Sulphonylurea/glinides	34 (31.1)	34 (32.7)	0.80^c^
α-glycosidase preparations	35 (34.0)	28 (25.5)	0.17 ^c^
FBG, median (IQR), mmol/L	8.46 (7.11-10.51)	8.46 (7.06-10.21)	0.85^b^
HbA_1_c, median (IQR), %	7.10 (6.50-8.50)	7.20 (6.60-8.31)	0.69^b^
Fasting insulin, median (IQR), uU/ml	8.46 (5.06-12.7)	8.8 (5.78-13.35)	0.46^b^
Fasting c-peptide, median (IQR), nmol/L	0.64 (0.51-0.95)	0.75 (0.55-0.92)	0.24^b^
HOMA-IR, median (IQR), units	1.80 (1.30-2.60)	2.00 (1.40-2.50)	0.38^b^
TG, median (IQR), mmol/L	1.56 (1.13-2.27)	1.65 (1.15-2.17)	0.82^b^
TC, mean (SD), mmol/L	4.93 (1.12)	4.96 (1.06)	0.88^a^
HDL, mean (IQR), mmol/L	1.57 (0.99-2.02)	1.61 (1.19-2.12)	0.33^b^
LDL, mean (SD), mmol/L	2.39 (1.10)	2.37 (0.96)	0.93^a^
VLDL, median (IQR), mmol/L	0.71 (0.51-1.03)	0.75 (0.52-0.99)	0.82^b^
Blood pressure, mean (SD), mmHg			
Systolic	135.37 (21.05)	135.72 (19.37)	0.90^a^
Diastolic	76.98 (11.02)	76.55 (10.74)	0.77^a^

FBG, Fasting Blood Glucose; HbA_1c_, Glycated Hemoglobin A_1C_; HDL, High-Density Lipoprotein; LDL, Low-Density Lipoprotein; VLDL, Very Low-Density Lipoprotein; HOMA-IR, Homeostasis Model Assessment-estimated Insulin Resistance; TC, Total Cholesterol; TG, Triglycerides; SD, standard deviation; IQR, Interquartile ranges, Kolmogorov-Smirnov test confirmed the normal distribution of the dataset. Normally distributed continuous variables are presented as mean ± standard deviation; Non-normally distributed continuous variables are presented as median and interquartile range (IQR). ^a^P values according to Student’s t test, ^b^P values according to Mann-Whitney test; ^c^P values according to Pearson Chi-square test; significance: P<0.05.

### Primary outcome

**Figure 2:**
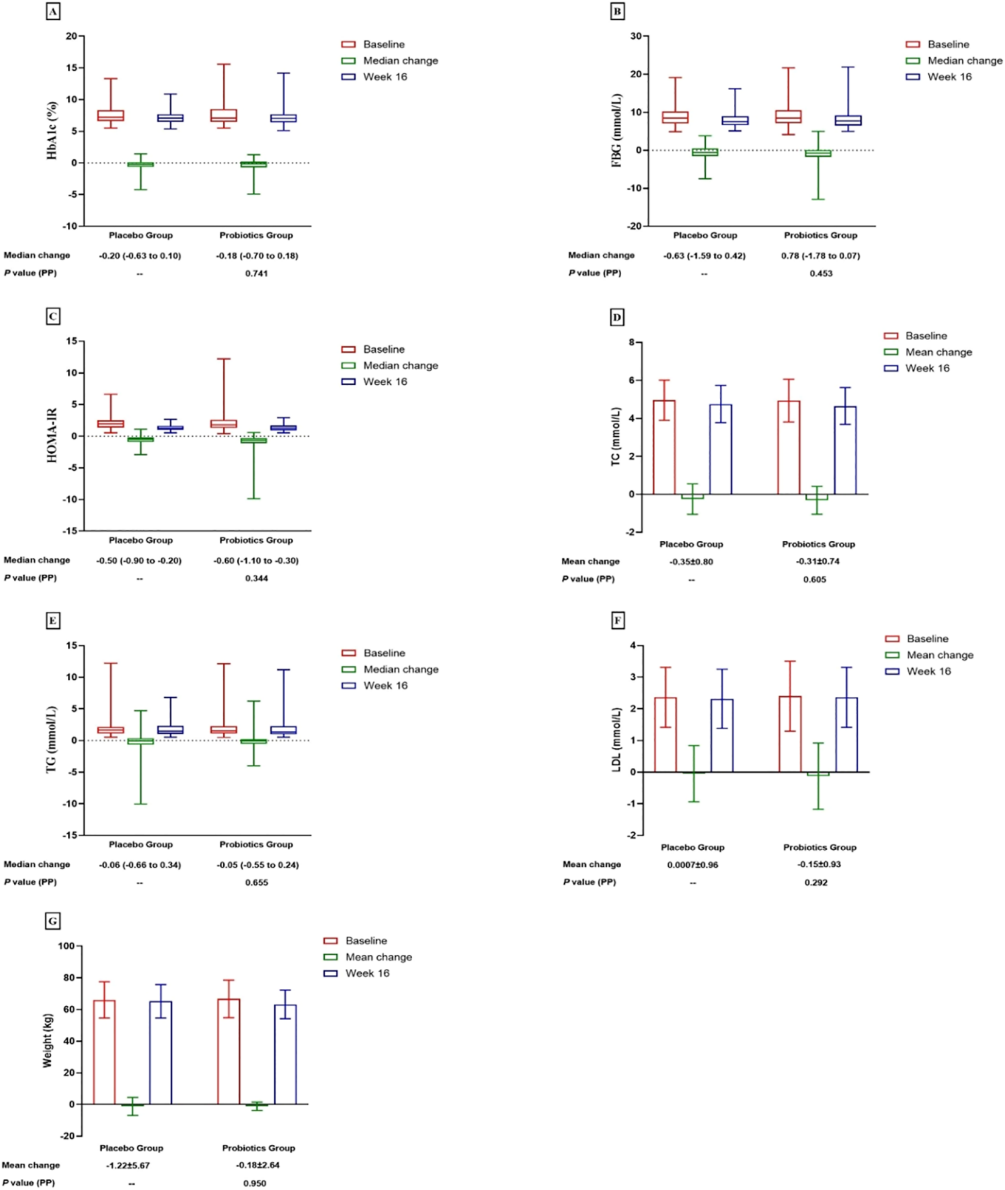
Changes in glucose parameters, lipid profiles and weight.

**Figure 3:**
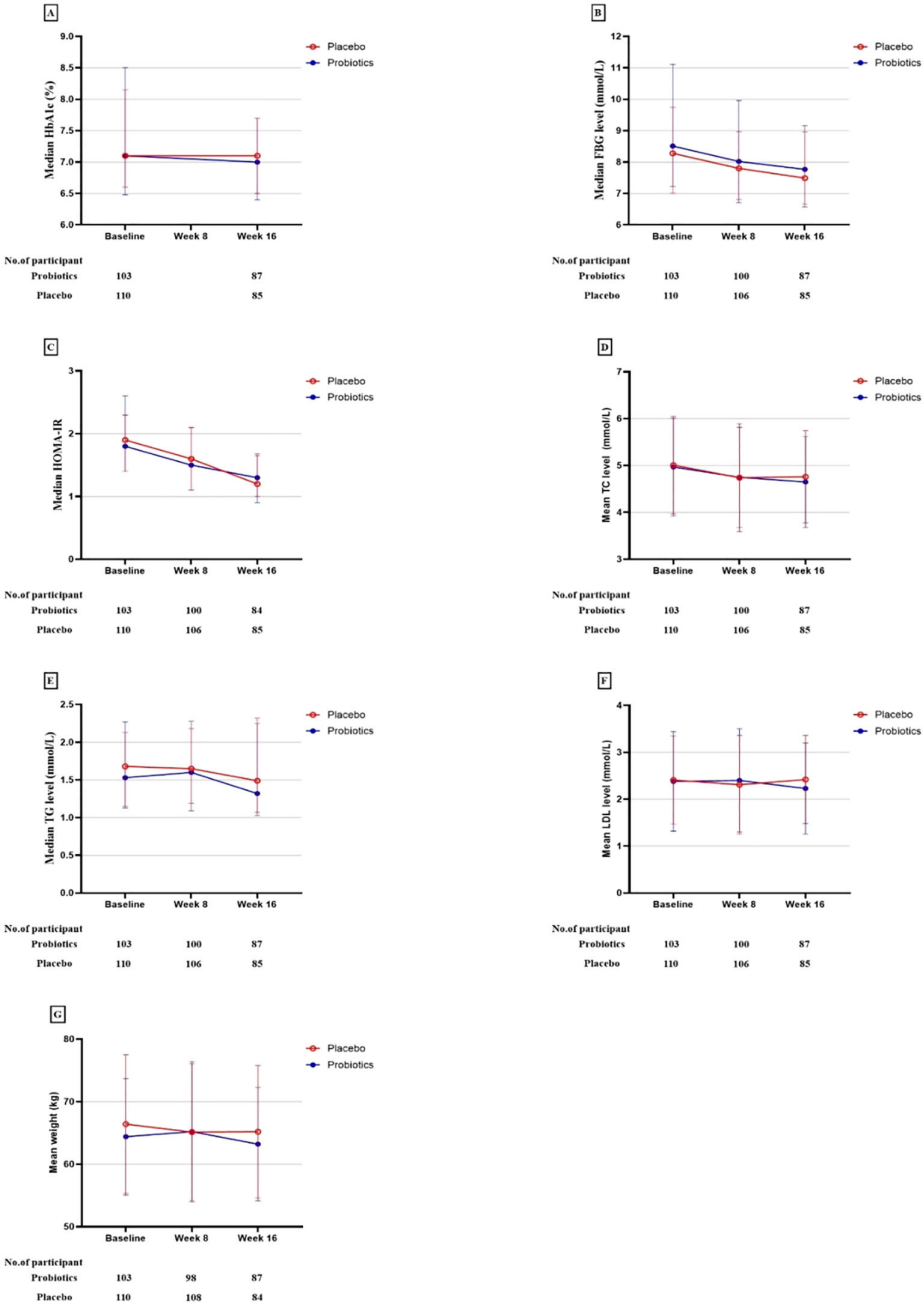
Primary and secondary study outcomes at baseline and while taking probiotic supplementation.

The probiotic, probiotic, and placebo groups exhibited a reduction in HbA1c and FBG levels after 16 weeks of intervention. However, in the ITT analysis, there were no statistically significant differences between the two groups in the changes in these glycemic-related parameters, indicating that the probiotics had no beneficial effect on glycemic control (**Table 2**, **Figures 2**, **3**).

**Table 2:** Changes of variable throughout study in probiotics and placebo group.

	Probiotics group (n =103)		Placebo group (n = 110)	ITT (*p*-value)^#^
	Mean Change (95%CI)	Differences of the mean changes (probiotics vs placebo) (95% CI)	Mean Change (95%CI)	
Primary Outcomes				
**HbA_1c_ %**				
Change at 16 weeks (95%CI)	**-0.44 (-0.66 to -0.22) ^&^**	-0.12 (-0.42 to 0.18)	**-0.33 (-0.52 to -0.15) ^&^**	0.941
**FBG (mmol/L)**				
Change at 8 weeks (95%CI)	-0.54 (-1.07 to 0.00)	0.25 (-0.55 to 1.06)	**-0.62 (-1.10 to -0.14) ^&^**	0.53
Change at 16 weeks (95%CI)	**-0.97 (-1.49 to -0.46) ^&^**	0.29 (-0.40 to 0.98)	**-0.90 (-1.32 to -0.47) ^&^**	0.41
Secondary Outcomes				
**Fasting c-peptide (nmol/L)**				
Change at 8 weeks (95%CI)	0.06 (-0.26 to 0.39)	-0.14 (-0.45 to 0.18)	**-0.11 (-0.16 to -0.55) ^&^**	0.40
Change at 16 weeks (95%CI)	**-0.22 (-0.28 to -0.17) ^&^**	-0.02 (-0.083 to 0.42)	**-0.24 (-0.29 to -0.18) ^&^**	0.53
**HOMA-IR**				
Change at 8 weeks (95%CI)	0.32 (-1.08 to 1.73)	0.35 (-1.03 to 1.73)	-0.13 (-0.59 to 0.32)	0.62
Change at 16 weeks (95%CI)	**-0.77 (-1.02 to -0.52) ^&^**	-0.03 (-0.19 to 0.13)	**-0.71 (-0.87 to -0.54) ^&^**	0.72
**Fasting insulin (uU/ml)**				
Change at 8 weeks (95%CI)	-0.39 (-1.84 to 1.07)	-2.60 (-8.22 to 3.02)	-1.71 (-3.96 to 0.53)	0.37
Change at 16 weeks (95%CI)	**-3.35 (-5.62 to -1.08) ^&^**	-1.27 (-4.89 to 2.35)	**-6.00 (-10.99to -1.02) ^&^**	0.49
**TG (mmol/L)**				
Change at 8 weeks (95%CI)	-0.22 (-0.47 to 0.03)	-0.08 (-0.47 to 0.31)	-0.03 (-0.32 to 0.26)	0.68
Change at 16 weeks (95%CI)	-0.12 (-0.44 to 0.19)	0.16 (-0.28 to 0.60)	-0.17 (-0.48 to 0.14)	0.48
**TC (mmol/L)**				
Change at 8 weeks (95%CI)	**-0.15 (-0.30 to -0.01) ^&^**	0.04 (-0.26 to 0.34)	**-0.22 (-0.37 to -0.06) ^&^**	0.80
Change at 16 weeks (95%CI)	**-0.28 (-0.46 to -0.09) ^&^**	-0.10 (-0.39 to 0.19)	**-0.19 (-0.38 to -0.015) ^&^**	0.48
**HDL (mmol/L)**				
Change at 8 weeks (95%CI)	-0.11 (-0.28 to 0.05)	-0.15 (-0.31 to 0.01)	-0.05 (-0.19 to 0.09)	0.07
Change at 16 weeks (95%CI)	-0.07 (-0.21 to 0.07)	0.01 (-0.13 to 0.15)	-0.17 (-0.33 to 0.00)	0.89
**LDL (mmol/L)**				
Change at 8 weeks (95%CI)	0.04 (-0.15 to 0.23)	0.12 (-0.17 to 0.41)	-0.07 (-0.24 to 0.11)	0.42
Change at 16 weeks (95%CI)	-0.16 (-0.36 to 0.05)	-0.19 (-0.47 to 0.09)	0.04 (-0.16 to 0.25)	0.20
**VLDL (mmol/L)**				
Change at 8 weeks (95%CI)	-0.11 (-0.22 to 0.00)	-0.05 (-0.22 to 0.13)	-0.01 (-0.14 to 0.19)	0.59
Change at 16 weeks (95%CI)	-0.06 (-0.19 to 0.09)	0.07 (-0.13 to 0.27)	-0.08 (-0.22 to 0.06)	0.47
**Weight (kg)**				
Change at 8 weeks (95%CI)	**-1.44 (-2.41 to -0.48) ^&^**	0.24 (-2.78 to 3.26)	**-1.08 (-1.98 to -0.19) ^&^**	0.88
Change at 16 weeks (95%CI)	**-3.43 (-5.10 to -1.77) ^&^**	-1.97 (-4.91 to 0.97)	-0.87 (-2.47 to 0.74)	0.19

FBG, fasting blood glucose; HbA1c, glycated hemoglobin; HDL, high-density lipoprotein; LDL, low-density lipoprotein; VLDL, very low-density lipoprotein; HOMA-IR, homeostasis model assessment-estimated insulin resistance; TC, total cholesterol; TG, triglycerides; ITT, intention-to-treat; SD, standard deviation; IQR, interquartile ranges. ^#^adjusting for age, sex, and the use of specific medications (Insulin, Metformin, sulphonylurea/glinide, α-glycosidase preparations); blod values^&^: P<0.05.

### Secondary outcomes

The study also observed reductions in the weight and serum levels of fasting insulin, HOMA-IR, fasting C-peptide, HOMA-IR, fasting C-peptide, and TC from baseline to the end of the 16-week intervention period in both groups. Other lipid parameters, including triglycerides (TG), high-density lipoprotein (HDL), low-density lipoprotein (LDL), and very-low-density lipoprotein (VLDL) showed no statistically significant changes within the group following the intervention. Nonetheless, the differences in these reductions between the probiotic and placebo groups were not significant in either analysis type, indicating that probiotics did not have a beneficial impact on lipid profiles and weight (**Table 2**, **Figures 2**, **3**).

### Adherence and adverse events

The adherence rates to the probiotic regimen were comparable to the placebo. Adverse events reported during the study were similarly distributed between the groups, indicating a comparable safety profile. No significant differences in the incidence of adverse events were noted, underscoring the similar tolerability of probiotic and placebo treatments.

## Discussion

Our study demonstrated that, following a 16-week intervention, probiotics elicited significant effects on fasting blood glucose, glycated hemoglobin, triglycerides (TC), and weight in patients with diabetes. However, these effects were not statistically significant when compared to the control group. Additionally, no discernible decrease was observed in other lipid parameters, including TG, high-density lipoprotein (HDL), and very-low-density lipoprotein (VLDL).

### Comparison with other studies

Our findings align with those of Barengolts et al. (25), who conducted a systematic review and meta-analysis of nine randomized controlled trials and found no significant effect of a 12-week probiotic intervention on fasting blood glucose or other diabetes-related factors. In contrast, a myriad of studies have presented divergent results, with several reporting significant reductions in serum HbA1c, FBG, and fasting insulin levels in T2DM patients following probiotic supplementation (25–28). Specifically, Raygan et al. (26) observed beneficial effects on glycemic control and HDL cholesterol levels after a 12-week regimen of diverse probiotics in patients with diabetes with concurrent coronary heart disease. Nonetheless, the Raygan (26) study had a small sample size of 60 patients, which may limit the generalizability of their findings. Some studies found no significant effect on HDL cholesterol or triglycerides (29), whereas others reported reductions in triglyceride levels (30, 31). The effect of probiotics on lipid profiles may depend on factors such as baseline cholesterol levels, treatment duration, and specific probiotic strains (29). These findings suggest that probiotics could be a potential adjunctive treatment for dyslipidemia; however, further research is needed to clarify their long-term effects and interactions with drug therapies (31). Furthermore, post-placebo intervention results in other studies indicated significant increases in fasting blood glucose and lipid levels, exacerbating the perceived effects of probiotics (26). The current study noted no significant improvement in body weight, consistent with prior findings that probiotic supplementation does not lead to notable weight loss. Typically, weight loss is more difficult in individuals with diabetes, especially when it coincides with enhanced glycemic control (32). In cases of poorly controlled diabetes, any improvement in glycemic regulation may decrease overall energy expenditure, thereby exacerbating the challenges associated with weight loss.

### Analysis of ineffectiveness

The lack of significant improvements in glycemic control and lipid profiles in our study may be attributed to several factors. First, the physical form of probiotics: liquid probiotics are more susceptible to external influences such as temperature and light exposure during storage, which could lead to their inactivation (33). Compared to solid storage, the internal environment of colonies in liquid storage is more vulnerable to external conditions, potentially affecting the growth and survival of probiotics (34). Additionally, nutrients may more easily diffuse out of the cells in liquid culture media, leading to nutrient deficiencies within the cells, and consequently, probiotic death (34). Furthermore, the stability of cell membranes may be compromised during liquid storage, resulting in probiotic inactivation (35). Studies have also indicated that the beneficial effects of probiotics on cholesterol levels are predominantly observed when probiotics are consumed in conjunction with dairy products. Consequently, liquid probiotics without any substrate may demonstrate reduced cell viability after oral administration, leading to suboptimal glucose and lipid control in T2DM patients.

Second, utilization of a single probiotic strain: Previous clinical trials employing two or more strains of probiotics reported significant reductions in glucose and lipid profiles (36–38). For example, Raygan et al. (26) found that a 12-week intake of probiotics containing Bifidobacterium, Lactobacillus casei, and Lactobacillus acidophilus significantly reduced the glycemic parameters in the intervention group. Similarly, Mohamadshahi et al. (28) reported that probiotic yogurt containing Lactobacillus acidophilus La-5 and Bifidobacterium lactis Bb-12 affects blood lipids. Various studies have demonstrated that probiotic supplements with diverse compositions have beneficial effects on glycemic control in patients with diabetes. Therefore, exploring the most effective probiotic compositions for T2DM patients in the future is imperative.

Third, dietary interference: The composition of the gut microbiota in different ethnic groups can be influenced by factors such as dietary habits, geographic locations, and genetics. Thus, the efficacy of probiotic supplements may vary, depending on the background microbiota composition of the population (39, 40). A diverse diet can provide essential nutrients for the intestine and promote the growth of probiotics. However, the relatively simplistic dietary structure of the Chinese people, characterized by high consumption of sugar, fat, and protein, might affect the types and quantities of probiotics, leading to their ineffectiveness and poor control of glucose and lipids. Recent evidence indicates that T2DM patients exhibit varying degrees of gut microbiota dysbiosis (41). Therefore, enhancing the gut microbiota through various methods, including diet control, regular exercise, healthy lifestyle, and taking probiotic supplements with suitable strains and doses, is crucial. Reducing the intake of high-sugar, high-salt, and high-fat foods, along with consuming foods rich in dietary fiber such as vegetables, fruits, and whole grains, can promote the growth and reproduction of intestinal probiotics. Additionally, improving sleep, regular meals, reasonable exercise, and stress reduction can improve the intestinal environment and promote the gut microbiota balance.

Fourth, we found a difference in the initial use of metformin between the two groups. Previous systematic reviews and retrospective studies have shown that the combination of metformin and probiotics can lower fasting blood glucose and glycated hemoglobin levels in patients to a greater extent than metformin alone. However, the meta-analysis did not incorporate the probiotic *Lactobacillus (* 42). Therefore, the potential combined effect of *Lactobacillus* and metformin in the treatment of patients with diabetes mellitus remains uncertain.

### Why placebo was effective

Our study observed partial improvements in blood glucose and lipid levels in the placebo group, comparable to the effects seen with probiotics, leading to no statistically significant difference between the two groups. This phenomenon might be attributed to the placebo effect (43), as the placebo shared a similar taste and odor with the probiotic treatment, potentially leading patients to subconsciously believe in their condition’s improvement due to the attention and care they received. Another contributing factor could be the use of low-concentration inactivated bacteria in the placebo. Research indicates that the metabolites or structural components of probiotics can be beneficial to the human body (44), and previous studies have shown that inactivated probiotics may play a role in weight reduction and cholesterol lowering, potentially through modulation of gut microbiota (45). Furthermore, peptidoglycan, a component of bacterial cell walls, may be instrumental in the regulation of GLP-1 secretion, enhancement of insulin sensitivity, and improvement of glucose tolerance (46). Thus, the therapeutic potential of dead bacteria in the treatment of diabetes should not be dismissed, and future studies should consider including groups that receive dead bacteria along with blank control groups to further explore this possibility.

### Clinical and research implications

The high costs of transporting and storing probiotics, coupled with their relatively insignificant therapeutic effects, have reduced the viability of using probiotics for diabetes treatment (47). This study provides a new perspective for clinical therapy, suggesting that inactivated bacteria might offer therapeutic outcomes comparable to those of live probiotics in the treatment of diabetes. Historically, the substantial costs associated with the transportation and storage of probiotics have been disproportionate to their therapeutic benefits, thereby limiting the practicality of their clinical use. However, employing inactivated bacteria could significantly mitigate this issue, making probiotic therapy for diabetes a more feasible option. Thus, this study offers important insights for future research offers important insights for future studies. Additionally, the potential effectiveness of dead bacteria in treating diabetes should not be overlooked. Future research should consider including groups treated with dead bacteria and blank control groups to further investigate the therapeutic effects of both probiotics and inactivated bacteria. Moreover, given the complexity of the gut microbiota, a 12-week intervention with probiotics may not effectively alter the gut microenvironment. Although previous foundational research (48) has shown the potential of probiotics to modify the microbiome, the required dosages often exceed practical limits for human use, and their long-term safety remains uncertain (49). Further research is required to determine whether low-dose inactivated bacteria produce similar therapeutic effects.

### Strengths and limitations

This study had several notable strengths. First, it was designed as a double-blind, randomized controlled trial to ensure a high level of scientific rigor. Additionally, the intervention period was relatively extended, spanning 16 weeks, which provided ample time to observe potential effects. This is the first study to use inactivated Lactobacillus as a placebo. However, our study has certain limitations. First, the short follow-up period was a limitation of this study, which was constrained by funding and patient acceptance. Another limitation of this study was the statistical difference in the use of diabetic medications between the two groups at baseline, which may be due to the small sample size. Third, as this was a single-center study, the results may not be broadly applicable to other T2DM patient populations, necessitating further research across multiple centers for a more comprehensive understanding of the impact of probiotic probiotics on diabetes. Another significant limitation was the lack of measurement of fecal bacterial loads and changes in short-chain fatty acid levels, which restricted our ability to thoroughly understand the mechanisms by which probiotics might influence glycemic control and insulin resistance. Moreover, although we adjusted for factors such as sex, age, smoking, and alcohol consumption, the presence of multiple comorbidities and the concomitant use of various medications among the elderly, which may interact with probiotics, limits the generalizability of our findings. In comparison, our study used only a single probiotic strain, which may have been inadequate owing to the complexity of the gut microbiota. In addition, the absence of a blank control group, which was initially omitted due to ethical considerations, is a notable design limitation.

## Conclusion

In conclusion, our 16-week intervention study demonstrated that probiotic supplementation had some effects on fasting blood glucose, glycated hemoglobin, triglycerides, and weight in patients with type 2 diabetes. However, these effects did not reach statistical significance when compared to the control group, and no clear improvements were observed in other lipid parameters. While we adjusted for several confounding factors, the study had limitations including its short duration, single-center design, and use of a single probiotic strain. The utility of probiotics as an alternative approach for diabetes management remains uncertain. Further research involving longer follow-up periods, multiple centers, diverse subject groups, and a variety of probiotic strains is needed to comprehensively assess the efficacy of probiotic supplementation in type 2 diabetes management.

## Data Availability

The raw data supporting the conclusions of this article will be made available by the authors, without undue reservation.

## References

[1] Saeedi P Petersohn I Salpea P Malanda B Karuranga S Unwin N. Global and regional diabetes prevalence estimates for 2019 and projections for 2030 and 2045: results from the international diabetes federation diabetes atlas, 9(Th) edition. Diabetes Res Clin Pract. (2019) 157:107843. doi: 10.1016/j.diabres.2019.107843 31518657

[2] Zhang Y Pan XF Chen J Xia L Cao A Zhang Y. Combined lifestyle factors and risk of incident type 2 diabetes and prognosis among individuals with type 2 diabetes: A systematic review and meta-analysis of prospective cohort studies. Diabetologia. (2020) 63:21–33. doi: 10.1007/s00125-019-04985-9 31482198

[3] Lalles JP. Microbiota-host interplay at the gut epithelial level, health and nutrition. J Anim Sci Biotechno. (2016) 7:66. doi: 10.1186/s40104-016-0123-7 PMC5101664 27833747

[4] Jandhyala SM Talukdar R Subramanyam C Vuyyuru H Sasikala M Nageshwar Reddy D. Role of the normal gut microbiota. World J Gastroenterol. (2015) 21:8787–803. doi: 10.3748/wjg.v21.i29.8787 PMC4528021 26269668

[5] O’Hara AM Shanahan F. The gut flora as a forgotten organ. EMBO Rep. (2006) 7:688–93. doi: 10.1038/sj.embor.7400731 PMC1500832 16819463

[6] Cani PD Delzenne NM. The role of the gut microbiota in energy metabolism and metabolic disease. Curr Pharm Des. (2009) 15:1546–58. doi: 10.2174/138161209788168164 19442172

[7] Cani PD Osto M Geurts L Everard A. Involvement of gut microbiota in the development of low-grade inflammation and type 2 diabetes associated with obesity. Gut Microbes. (2012) 3:279–88. doi: 10.4161/gmic.19625 PMC3463487 22572877

[8] Le Chatelier E Nielsen T Qin J Prifti E Hildebrand F Falony G. Richness of human gut microbiome correlates with metabolic markers. Nature. (2013) 500:541–6. doi: 10.1038/nature12506 23985870

[9] van Olden C Groen AK Nieuwdorp M. Role of intestinal microbiome in lipid and glucose metabolism in diabetes mellitus. Clin Ther. (2015) 37:1172–7. doi: 10.1016/j.clinthera.2015.03.008 25922340

[10] Frost F Kacprowski T Ruhlemann M Pietzner M Bang C Franke A. Long-term instability of the intestinal microbiome is associated with metabolic liver disease, low microbiota diversity, diabetes mellitus and impaired exocrine pancreatic function. Gut. (2021) 70:522–30. doi: 10.1136/gutjnl-2020-322753 PMC7873430 33168600

[11] Martin FP Wang Y Sprenger N Yap IK Lundstedt T Lek P. Probiotic modulation of symbiotic gut microbial-host metabolic interactions in a humanized microbiome mouse model. Mol Syst Biol. (2008) 4:157. doi: 10.1038/msb4100190 18197175 PMC2238715

[12] Li G Feng H Mao XL Deng YJ Wang XB Zhang Q. The effects of probiotics supplementation on glycaemic control among adults with type 2 diabetes mellitus: A systematic review and meta-analysis of randomised clinical trials. J Transl Med. (2023) 21:442. doi: 10.1186/s12967-023-04306-0 37415167 PMC10324246

[13] Paul P Kaul R Harfouche M Arabi M Al-Najjar Y Sarkar A. The effect of microbiome-modulating probiotics, prebiotics and synbiotics on glucose homeostasis in type 2 diabetes: A systematic review, meta-analysis, and meta-regression of clinical trials. Pharmacol Res. (2022) 185:106520. doi: 10.1016/j.phrs.2022.106520 36272640

[14] Andersson U Bränning C Ahrné S Molin G Alenfall J Onning G. Probiotics lower plasma glucose in the high-fat fed C57bl/6j mouse. Benef Microbes. (2010) 1:189–96. doi: 10.3920/bm2009.0036 21840806

[15] Zavišić G Ristić S Rikalović M Petković B Janković D Vukadinović A. Beneficial effects of probiotic supplementation on glucose and triglycerides in a mouse model of metabolic syndrome. J Funct Foods. (2022) 95:105167. doi: 10.1016/j.jff.2022.105167

[16] Asgharzadeh F Tanomand A Ashoori MR Asgharzadeh A Zarghami N. Investigating the effects of lactobacillus casei on some biochemical parameters in diabetic mice. J Endocrinology Metab Diabetes South Afr. (2017) 22:47–50. doi: 10.1080/16089677.2017.1378460

[17] Yao K Zeng L He Q Wang W Lei J Zou X. Effect of probiotics on glucose and lipid metabolism in type 2 diabetes mellitus: A meta-analysis of 12 randomized controlled trials. Med Sci Monit. (2017) 23:3044–53. doi: 10.12659/msm.902600 PMC5491138 28638006

[18] Razmpoosh E Javadi A Ejtahed HS Mirmiran P Javadi M Yousefinejad A. The effect of probiotic supplementation on glycemic control and lipid profile in patients with type 2 diabetes: A randomized placebo controlled trial. Diabetes Metab Syndr. (2019) 13:175–82. doi: 10.1016/j.dsx.2018.08.008 30641692

[19] He J Zhang F Han Y. Effect of probiotics on lipid profiles and blood pressure in patients with type 2 diabetes: A meta-analysis of rcts. Med (Baltimore). (2017) 96:e9166. doi: 10.1097/md.0000000000009166 PMC5758152 29390450

[20] Mazloom Z Yousefinejad A Dabbaghmanesh MH. Effect of probiotics on lipid profile, glycemic control, insulin action, oxidative stress, and inflammatory markers in patients with type 2 diabetes: A clinical trial. Iran J Med Sci. (2013) 38:38–43. 23645956 PMC3642943

[21] Ben Othman M Sakamoto K. Effect of inactivated bifidobacterium longum intake on obese diabetes model mice (Tsod). Food Res Int. (2020) 129:108792. doi: 10.1016/j.foodres.2019.108792 32036897

[22] Zaha DC Bungau S Aleya S Tit DM Vesa CM Popa AR. What antibiotics for what pathogens? The sensitivity spectrum of isolated strains in an intensive care unit. Sci Total Environ. (2019) 687:118–27. doi: 10.1016/j.scitotenv.2019.06.076 31207502

[23] Zhou J Lin Y Liu Y Chen K. Antibiotic exposure and risk of type 2 diabetes mellitus: A systematic review and meta-analysis. Environ Sci pollut Res Int. (2021) 28:65052–61. doi: 10.1007/s11356-021-16781-3 34622400

[24] Levy JC Matthews DR Hermans MP. Correct homeostasis model assessment (Homa) evaluation uses the computer program. Diabetes Care. (1998) 21:2191–2. doi: 10.2337/diacare.21.12.2191 9839117

[25] Barengolts E Smith ED Reutrakul S Tonucci L Anothaisintawee T. The effect of probiotic yogurt on glycemic control in type 2 diabetes or obesity: A meta-analysis of nine randomized controlled trials. Nutrients. (2019) 11 (3):671. doi: 10.3390/nu11030671 30897796 PMC6471569

[26] Raygan F Rezavandi Z Bahmani F Ostadmohammadi V Mansournia MA Tajabadi-Ebrahimi M. The effects of probiotic supplementation on metabolic status in type 2 diabetic patients with coronary heart disease. Diabetol Metab Syndr. (2018) 10:51. doi: 10.1186/s13098-018-0353-2 29946368 PMC6008939

[27] Ejtahed HS Mohtadi-Nia J Homayouni-Rad A Niafar M Asghari-Jafarabadi M Mofid V. Effect of probiotic yogurt containing lactobacillus acidophilus and bifidobacterium lactis on lipid profile in individuals with type 2 diabetes mellitus. J Dairy Sci. (2011) 94:3288–94. doi: 10.3168/jds.2010-4128 21700013

[28] Mohamadshahi M Veissi M Haidari F Javid AZ Mohammadi F Shirbeigi E. Effects of probiotic yogurt consumption on lipid profile in type 2 diabetic patients: A randomized controlled clinical trial. J Res Med Sci. (2014) 19:531–6. PMC4155708 25197295

[29] Cho YA Kim J. Effect of probiotics on blood lipid concentrations: A meta-analysis of randomized controlled trials. Med (Baltimore). (2015) 94:e1714. doi: 10.1097/md.0000000000001714 PMC4985374 26512560

[30] Zarezadeh M Musazadeh V Faghfouri AH Roshanravan N Dehghan P. Probiotics act as a potent intervention in improving lipid profile: an umbrella systematic review and meta-analysis. Crit Rev Food Sci Nutr. (2023) 63:145–58. doi: 10.1080/10408398.2021.2004578 34817299

[31] Gadelha C Bezerra AN. Effects of probiotics on the lipid profile: systematic review. J Vasc Bras. (2019) 18:e20180124. doi: 10.1590/1677-5449.180124 31447899 PMC6690648

[32] Franz MJ VanWormer JJ Crain AL Boucher JL Histon T Caplan W. Weight-loss outcomes: A systematic review and meta-analysis of weight-loss clinical trials with a minimum 1-year follow-up. J Am Diet Assoc. (2007) 107:1755–67. doi: 10.1016/j.jada.2007.07.017 17904936

[33] Ongol MP Sawatari Y Ebina Y Sone T Tanaka M Tomita F. Yogurt fermented by lactobacillus delbrueckii subsp. Bulgaricus H+ -atpase-defective mutants exhibits enhanced viability of bifidobacterium breve during storage. Int J Food Microbiol. (2007) 116:358–66. doi: 10.1016/j.ijfoodmicro.2007.02.019 17434219

[34] Shah NP. Probiotic bacteria: selective enumeration and survival in dairy foods. J Dairy Sci. (2000) 83:894–907. doi: 10.3168/jds.S0022-0302(00)74953-8 10791807

[35] Shori AB. The potential applications of probiotics on dairy and non-dairy foods focusing on viability during storage. Biocatalysis Agric Biotechnol. (2015) 4:423–31. doi: 10.1016/j.bcab.2015.09.010

[36] Sun J Buys N. Effects of probiotics consumption on lowering lipids and cvd risk factors: A systematic review and meta-analysis of randomized controlled trials. Ann Med. (2015) 47:430–40. doi: 10.3109/07853890.2015.1071872 26340330

[37] Razmpoosh E Javadi M Ejtahed HS Mirmiran P. Probiotics as beneficial agents in the management of diabetes mellitus: A systematic review. Diabetes Metab Res Rev. (2016) 32:143–68. doi: 10.1002/dmrr.2665 25963407

[38] Zhang Q Wu Y Fei X. Effect of probiotics on glucose metabolism in patients with type 2 diabetes mellitus: A meta-analysis of randomized controlled trials. Medicina (Kaunas). (2016) 52:28–34. doi: 10.1016/j.medici.2015.11.008 26987497

[39] Senghor B Sokhna C Ruimy R Lagier J-C. Gut microbiota diversity according to dietary habits and geographical provenance. Hum Microbiome J. (2018) 7-8:1–9. doi: 10.1016/j.humic.2018.01.001

[40] Conlon MA Bird AR. The impact of diet and lifestyle on gut microbiota and human health. Nutrients. (2014) 7:17–44. doi: 10.3390/nu7010017 25545101 PMC4303825

[41] Que Y Cao M He J Zhang Q Chen Q Yan C. Gut bacterial characteristics of patients with type 2 diabetes mellitus and the application potential. Front Immunol. (2021) 12:722206. doi: 10.3389/fimmu.2021.722206 34484230 PMC8415158

[42] Memon H Abdulla F Reljic T Alnuaimi S Serdarevic F Asimi ZV. Effects of combined treatment of probiotics and metformin in management of type 2 diabetes: A systematic review and meta-analysis. Diabetes Res Clin Pract. (2023) 202:110806. doi: 10.1016/j.diabres.2023.110806 37369280

[43] Arnstein P. The placebo effect. Semin Integr Med. (2003) 1:125–35. doi: 10.1016/S1543-1150(03)00026-7

[44] Ross RP Mills S Hill C Fitzgerald GF Stanton C. Specific metabolite production by gut microbiota as a basis for probiotic function. Int Dairy J. (2010) 20:269–76. doi: 10.1016/j.idairyj.2009.12.003

[45] Vallianou N Stratigou T Christodoulatos GS Tsigalou C Dalamaga M. Probiotics, prebiotics, synbiotics, postbiotics, and obesity: current evidence, controversies, and perspectives. Curr Obes Rep. (2020) 9:179–92. doi: 10.1007/s13679-020-00379-w 32472285

[46] Williams L Alshehri A Robichaud B Cudmore A Gagnon J. The role of the bacterial muramyl dipeptide in the regulation of glp-1 and glycemia. Int J Mol Sci. (2020) 21 (15):5252. doi: 10.3390/ijms21155252 32722085 PMC7432949

[47] Terpou A Papadaki A Lappa IK Kachrimanidou V Bosnea LA Kopsahelis N. Probiotics in food systems: significance and emerging strategies towards improved viability and delivery of enhanced beneficial value. Nutrients. (2019) 11 (7):1591. doi: 10.3390/nu11071591 31337060 PMC6683253

[48] Salminen SJ Gueimonde M Isolauri E. Probiotics that modify disease risk. J Nutr. (2005) 135:1294–8. doi: 10.1093/jn/135.5.1294 15867327

[49] Sanders ME Akkermans LM Haller D Hammerman C Heimbach J Hörmannsperger G. Safety assessment of probiotics for human use. Gut Microbes. (2010) 1:164–85. doi: 10.4161/gmic.1.3.12127 PMC3023597 21327023

