# Supplementary material for: Effect of probiotics on glycemic control and lipid profiles in patients with type 2 diabetes mellitus: a randomized, double blind, controlled trial
Source: Front Endocrinol (Lausanne). 2024 Sep 16;15:1440286. doi: 10.3389/fendo.2024.1440286 (PMC11439702; doi:10.3389/fendo.2024.1440286)
Supplement: Supplementary file 2 [file Table1.docx]

**sTable1**

| Domain | Indicators/Tools | Baseline | week 8 | week 16 |
| --- | --- | --- | --- | --- |
| Demographics | Age, gender, | X | - | - |
| Clinical characteristics | Blood pressure | X | - | - |
| Diabetes | HbA_1c_ | X | X | X |
|  | HOMA-IR | X | X | X |
|  | Fasting insulin | X | X | X |
|  | Fasting c-peptide | X | X | X |
|  | FBG | X | X | X |
|  | Background diabetes therapy | X | X | X |
| Weight |  | X | X | X |
| Lipidemia | TG | X | X | X |
|  | TC | X | X | X |
|  | HDL | X | X | X |
|  | LDL | X | X | X |
|  | VLDL | X | X | X |

**Overview of assessment during the study**

*HbA1c*: glycated hemoglobin; *HOMA-IR*: homeostasis model assessment-estimated insulin resistance; *FBG*: fasting blood glucose, *TG*: triglycerides; *TC*: total cholesterol; *HDL*: high-density lipoprotein, *LDL*: low-density lipoprotein, *VLDL*: very low-density lipoprotein,
